# Role of articaine and perineural dexamethasone in prolonging postoperative analgesia in mandibular third molar surgery: A comparative analysis

**DOI:** 10.4317/medoral.27891

**Published:** 2026-01-24

**Authors:** Abdullah Tolga Şitilci, Berkem Atalay, Tuğba Kuşlu 3, Özen Doğan Onur

**Affiliations:** 1 Department of Oral and Maxillofacial Surgery, Faculty of Dentistry, Istanbul University, Istanbul, Turkey; 2 Vocational School of Health Sciences, Istanbul University-Cerrahpasa, Istanbul, Turkey; 3 Private Practice, Istanbul, Turkey

## Abstract

**Background:**

Pain management in dentistry, particularly in procedures such as mandibular third molar surgery, poses a significant challenge due to the transient efficacy of traditional local anesthetics. To address this, adjunctive therapies such as dexamethasone have been explored to prolong anesthesia and alleviate postoperative pain. However, the efficacy and safety of this approach in mandibular third molar surgery remain underexplored.

**Material and Methods:**

This study assessed the efficacy and safety of combining articaine with perineural dexamethasone for inferior alveolar nerve block in patients undergoing mandibular third molar extraction. Sixty patients aged 18-35 years were enrolled and randomly assigned to three groups receiving different anesthesia protocols. Anesthesia duration, postoperative pain scores, and adverse events were evaluated.

**Results:**

The incorporation of dexamethasone into articaine-based anesthesia significantly prolonged the duration of analgesia compared to articaine alone, underscoring its potential as an effective adjunctive therapy. While no significant differences were observed in the duration of operation, analgesic consumption, or incidence of adverse events among the groups, trends favoring the articaine-dexamethasone cohorts were noted in postoperative pain scores.

**Conclusions:**

Combining articaine with perineural dexamethasone for inferior alveolar nerve block prolongs postoperative analgesia in mandibular third molar surgery. While additional research using larger sample sizes and longer follow-up durations is needed, these findings imply that this combination could be beneficial for improving pain management during oral surgery.

## Introduction

The removal of wisdom teeth, particularly the mandibular third molars, is one of the most common procedures in oral and maxillofacial surgery. Although a routine operation, it is often associated with various complications that can affect recovery and comfort, such as pain, swelling, and trismus (1 , 2). The inflammatory response after a third molar extraction is quite complex, involving the release of chemical mediators such as prostaglandins, bradykinin, and cytokines (3). These substances contribute to swelling in local tissues, increased blood vessel permeability, and heightened pain sensitivity. Effectively managing these postoperative complications is essential to improve patient satisfaction and accelerate recovery. One way to do this is to develop and/or refine anesthesia techniques. The use of local anesthetics is essential for effective pain control during dental surgery. Articaine is one of the most valuable amide type local anesthetics and has gained considerable popularity in recent years due to its unique chemical structure, which incorporates a thiophene ring (4). This structural feature increases lipid solubility, enhancing the drug's ability to penetrate nerve membranes and dense osseous tissue such as the mandibular bone, a common challenge in achieving effective anesthesia for procedures involving the lower jaw. Despite these pharmacological advantages, articaine shares a limitation with other local anesthetics: Its duration of action is relatively short.

Steroids prolong the duration of nerve block in regional anesthesia. Glucocorticoids, when used as adjuvants to local anesthetic solutions, enhance block quality and extend anesthetic action. Dexamethasone, in particular, induces vasoconstriction, which slows local anesthetic uptake and absorption, thereby prolonging anesthesia and improving patient comfort. Beyond this pharmacological effect, dexamethasone is a potent anti inflammatory agent that suppresses the release of interleukins and cytokines while promoting anti inflammatory mediators. Consequently, both local and systemic administration of dexamethasone have been associated with reduced postoperative pain, extended anesthetic duration, and prevention of operative site edema, without significant adverse effects (5 , 6). Compared to methylprednisolone, dexamethasone demonstrates greater potency and a longer duration of action (7). A clinical study further indicated that dexamethasone injections significantly reduced pain and edema formation relative to controls, although they had only limited influence on trismus (8). Based on these properties, we hypothesized that combining articaine with dexamethasone for inferior alveolar nerve block would prolong postoperative analgesia. We investigated this, as well as the effects on postoperative pain and vital signs.

## Material and Methods

Study design

A prospective, randomized, controlled, clinical trial design was employed. The trial was conducted at Istanbul University Faculty of Dentistry. All patients received detailed information about the study and provided written informed consent.

Patient Involvement

Patients were not involved in the design of the study, selection of outcome measures, or implementation of the trial. During protocol development, the potential impact on patients and the practicality of follow-up procedures were evaluated.

Patients

We enrolled 60 patients between 18 and 35 years of age who were admitted to our clinic for mandibular third molar extraction in Class II, Position B, according to Pell and Gregory's classification. This system defines the degree to which the third molar is embedded in the vertical and horizontal dimensions and specifies its relation to the occlusal plane. Grades A-C describe the vertical dimension, while mandibular branches 1-3 describe the horizontal dimension (9).

Exclusion criteria included use of anti-inflammatory drugs within the previous 2 weeks, use of antibiotics within the previous 15 days, any systemic mental or physical health problems, contraindications to any component of the local anesthetic solution, or pregnancy or breastfeeding. Patients meeting these criteria were screened, and eligibility was confirmed prior to randomization.

Randomization and blinding

Block randomization was used to assign participants to groups. Equal numbers of group assignments were written on folded paper slips and placed in an opaque container. For each eligible participant, a slip was drawn immediately before anesthesia to determine treatment allocation. Both patients and the investigator responsible for postoperative assessments and data collection (AT) were blinded to group assignment. The surgeon (BA) performing the extraction was aware of the allocated intervention due to anesthetic preparation but did not participate in postoperative outcome assessment or data analysis.

Intervention

A trained nurse performed extraoral antisepsis with a 2% chlorhexidine gluconate solution and intraoral antisepsis with 15mL 0.12% chlorhexidine gluconate for 1min immediately before the operation. Anesthesia was administered as follows: In Group A, patients received 120mg 2% articaine with 2mL saline; in Group B, patients received 120mg 2% articaine with 4mg dexamethasone and 1mL saline; and in Group C, patients received 120mg 2% articaine with 8mg dexamethasone. Epinephrine (1:100,000) was added to all injections to ensure regional anesthetic block of the inferior alveolar and lingual nerves, as well as the buccal area near the third molar to be extracted. Dexamethasone doses were chosen based on previous studies reporting no significant safety concerns within the commonly studied dose range for peripheral nerve blocks, specifically 4mg and 8mg (10). All patients underwent surgery using the same technique performed by the same surgeon (BA). Patients were seated comfortably in a semi reclining position under medical observation, and monitoring devices were applied. Blood pressure, pulse, respiratory rate, and arterial oxygen saturation were measured before extraction, during the procedure, and at 2h after completion. Then all data were monitored and recorded by one investigator (AT).

First, a linear incision was made in the alveolus with a No. 15 scalpel, starting from the second molar in the buccal region; then an oblique 1-cm incision was made. When necessary, osteotomy was performed with a high speed rotary drill under saline irrigation. Then, after extraction, irregular bone margins were flattened, and the wound was sutured with No. 3-0 silk in all operations. Local anesthesia and surgery were applied to all patients by the same surgeon. Then the patients were managed with standard postoperative procedures. Cold compression with an ice pack was recommended for 10min at 10-min intervals every hour during the first 24h after surgery. The onset of anesthesia was defined as the time from injection until loss of sensation over the buccal gingiva of the tooth to be extracted. The duration of anesthesia was defined as the time from injection until the patient requested the first analgesic medication. All patients received a standardized postoperative analgesic regimen. Dexketoprofen trometamol (25mg) was prescribed as rescue analgesia and administered every 8h after the onset of pain. No additional or alternative analgesics were permitted during the first 24h postoperatively.

**Figure 1:**
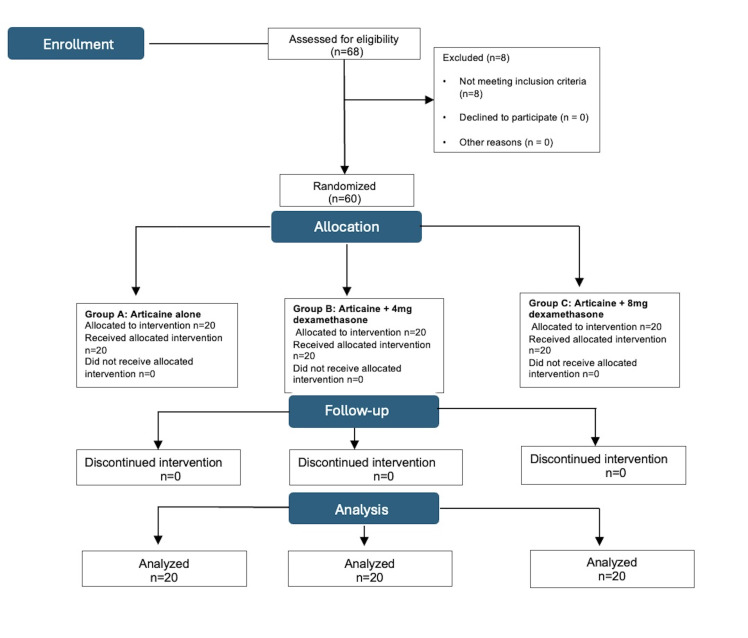
CONSORT flow diagram showing patient enrollment, randomization by folded-paper draw, group allocation, follow-up, and analysis. No patients were lost to follow-up, and all randomized participants were included in the final analysis.

Pain threshold was assessed using a probing method. Buccal and palatal soft tissue sensitivity over the premolar to be extracted was recorded using a visual analog scale (VAS) at 10min after injection and every 20min thereafter for 2h. The procedure was completed once adequate analgesia was assured. A 0.5-mg injectable atropine solution was readily available for administration in case of bradycardia, defined as a heart rate below 50 beats/min. The patients were discharged after stabilization, with clear post extraction care instructions. The investigator was responsible for contacting patients by phone the day after discharge to collect data on whether and when the first analgesic was needed. Figure1 shows patient flow during the study.

1Evaluation of Adverse Events

Neurological events, such as sensory deficits, motor weakness, paresthesia, numbness, dysesthesia, and other nerve injury symptoms, were assessed through patient interviews during a telephone follow-up on the first postoperative day. Systemic events were monitored by tracking blood pressure, heart rate, and oxygen saturation during surgery and for 2 h postoperatively in the consultation room to identify early systemic effects of dexamethasone, including hemodynamic instability. Early drug-related side effects, such as angioedema, urticaria, nausea, vomiting, and generalized weakness, were also monitored.

Outcome

Our primary hypothesis was that perineural dexamethasone would prolong the duration of analgesia compared to articaine alone. The primary outcome was the duration of postoperative analgesia, defined as the time from local anesthetic injection to the first rescue analgesic. Secondary outcomes included postoperative pain intensity (VAS), 24-h analgesic consumption, hemodynamic parameters, and safety outcomes.

Statistical analysis

A priori power analysis was performed using G*Power version 3.1. With an effect size of 0.52 for the primary outcome, alpha error probability of 0.05, and 80% power, a total sample size of 60 patients (20 per group) was considered sufficient.

Categorical variables are reported as counts and percentages (%), while continuous variables are reported as the mean with standard deviation (SD) or median and inter-quartile range in accordance with the distribution of the data. The Kolmogorov-Smirnov test was used to determine the normality of quantitative variables. One-way ANOVA was used to compare differences among groups in age, duration of analgesia, and duration of the operation. For the primary outcome (duration of analgesia), pairwise comparisons between groups were performed using Tukey's test to control for multiple comparisons. The Kruskal-Wallis test was used to compare differences among groups in the amount of analgesic taken in the first 24 h, the amount of analgesic taken in the first 24 h after pain onset, the time of onset, and the time at which the first analgesic was taken.

Pearson's chi-square test was used to compare the sex ratio among groups. Changes in systolic blood pressure (SBP), diastolic blood pressure (DBP), heart rate, and oxygen saturation before, during, and after the operation were compared between the groups using repeated-measures two-way ANOVA. Changes in VAS scores before vs. after the operation were compared between the groups via nonparametric analysis of longitudinal data in factorial experiments with the R package "npaRLD." The Pearson correlation coefficient or Spearman's rho was used to assess the relationships between the duration of analgesia and other numerical variables. All calculations were performed using R software (ver. 3.6.0 for Windows; R Development Core Team) or Jamovi (ver. 1.0.0; The Jamovi Project 2019). In statistical analyses, a p-value of 0.05 was deemed significant. All patients received their assigned intervention, completed the study protocol, and were included in the final analysis. Hence, the analyzed population represents both the intention-to-treat and per-protocol groups.

## Results

In all, 60 patients were included. Their mean ages were 24.6±5.6, 26.1±5.3, and 24.8±4.9 years in groups A, B, and C, respectively (p=0.647). The duration of analgesia was shortest in group A (174.3±15.8 min) and longest in group C (260.9±25.3 min) (p&lt;0.001). There were no significant differences (p&lt;0.001 unless stated otherwise) among the groups in the duration of operation, amount of analgesic consumed in the first 24 h, amount of analgesic consumed in the 24 h after the onset of pain, time of pain onset, or time of first analgesic taken (Table 1).

Table 1

One-way ANOVA showed a statistically significant difference in analgesia duration among the groups. Tukey's test indicated that duration was significantly longer in Group B than in Group A, and was significantly longer in Group C than in both Groups A and B. The mean differences in analgesia duration were approximately 36.6 min between Groups A and B (95% CI: 25-48 min), 50.0 min between Groups B and C (95% CI: 37-63min), and 86.6 min between Groups A and C (95% CI: 73-100 min). No cases of nerve injury, neurological deficit, or systemic or other adverse events were observed in any group.

No significant differences were observed between the mean pre- and postoperative DBPs of the patients, whereas there were differences in SBPs, heart rate, oxygen saturation, and VAS scores (Table 2); however, the p-values did not reach statistical significance for these measures.

Table 2

There were weak correlations among intraoperative heart rate, postoperative heart rate, postoperative oxygen saturation, and duration of analgesia across the entire study population. The postoperative DBP and duration of operation in Group A were moderately correlated with the duration of analgesia. Intraoperative and postoperative SBPs were weakly positively correlated with the duration of analgesia in Group C (Table 3).

Table 3

## Discussion

The duration of postoperative analgesia after extraction of the mandibular third molar was considerably extended by perineural administration of dexamethasone when combined with articaine for inferior alveolar nerve block. To reduce differences in surgical difficulty, only mandibular third molars classified as Pell and Gregory Class II, Position B were included. One experienced surgeon performed all procedures using the same technique, with identical flap design, osteotomy when required, and the same method for wound closure. This approach ensured that surgical trauma was comparable across study groups. Patients who received articaine alone experienced the shortest mean analgesia duration (174.3±15.8min), whereas those who received articaine with dexamethasone achieved the longest duration (260.9±25.3min). This significant difference demonstrates the beneficial effect of dexamethasone as an adjuvant. The longer pain free interval represents a clinically important advantage because it reduces the need for rescue medication and improves patient comfort in the immediate postoperative period. However, there were no statistically significant differences between groups in postoperative analgesic consumption, time to pain onset, or VAS scores. Pain was assessed only during the early postoperative period because the primary objective was to measure the duration of block related analgesia rather than the entire course of inflammatory pain following third molar surgery. Early postoperative pain depends primarily on the effectiveness and duration of local anesthetic blocks, so this period was chosen as the main outcome window.

In a previous study, a single submucosal injection of 4mg dexamethasone during surgery reduced facial edema on postoperative day 2 compared to controls; 8mg did not provide additional benefit, and the effects on pain and trismus were limited (11). By contrast, another randomized comparative study reported that dexamethasone administered intramuscularly (4mg) or submucosally (4mg) significantly decreased pain and swelling and improved patient reported quality of life, with the overall efficacy of the two routes being similar (12). In both these previous studies and in ours, variability in individual pain thresholds, surgical difficulty, postoperative analgesic protocols, and operator technique may have influenced analgesic demand, potentially masking differences between groups.

Two previous studies found that dexamethasone combined with articaine resulted in both longer analgesic duration and reduced postoperative pain after third molar surgery (13 , 14). Our results support those findings and imply that dexamethasone can effectively improve the performance of articaine. We found that local addition of dexamethasone significantly improved analgesia without causing any notable side effects. Compared to intravenous dexamethasone, perineural administration may provide better pain relief and longer anesthesia (15). Systematic reviews have shown that perineural dexamethasone significantly prolongs postoperative analgesia compared to intravenous administration as an adjuvant to peripheral nerve blocks. For example, one study reported that perineural dexamethasone extended analgesia by about 2.7 h relative to intravenous dexamethasone (16). These findings indicate that the local pharmacological effects of dexamethasone at the perineural site, rather than its systemic anti inflammatory action, are more critical for enhancing block efficacy.

We evaluated adverse events with an emphasis on systemic and neurological safety. Systemic effects were monitored intraoperatively and during the first 2 postoperative hours by continuously assessing vital signs, including systolic and diastolic blood pressure, heart rate, and oxygen saturation. Early structured monitoring is a strength of the study, as the potential systemic effects of dexamethasone, such as hemodynamic instability, angioedema, and urticaria, are most likely to occur during or soon after administration. Neurotoxicity is another potential concern with steroid use, particularly given the proximity to the nerve sheath. However, none of the participants in our study or in a previous study reported postoperative symptoms related to nerve injury (17). The absence of early neurological complications supports the short term safety of perineural dexamethasone at both 4-mg and 8-mg doses, although the lack of long term neurological follow up remains a limitation.

The optimal dose for perineural dexamethasone is still unclear. Previous randomized trials have assessed doses of 1-8 mg as adjuvants to peripheral nerve blocks. Meta analyses have reported no significant differences in safety outcomes between commonly used doses, such as 4 mg and 8 mg (10 , 18). In this study, higher dexamethasone doses prolonged postoperative analgesia, with Group C showing the longest duration. However, pain scores and analgesic consumption did not differ significantly between Groups B and C. These findings, consistent with Kirkham et al. (10), imply a ceiling effect, in which higher perineural doses may extend analgesia without additional clinical benefit. Given that the secondary pain outcomes were similar, lower doses may be sufficient to achieve optimal analgesic effect. Larger studies with longer follow up are needed to clarify the dose-response relationship and to determine whether higher doses provide benefits beyond prolonged early pain relief.

Articaine has superior lipid solubility and a faster onset of action. Thus, a dexamethasone-articaine mixture appears to be an appropriate solution for pain control (19). However, Stojanovi et al. (20) investigated the combination of dexamethasone with ropivacaine and reported a significant extension of inferior alveolar nerve block duration. Despite the use of a different anesthetic agent, the consistent results support the theory that dexamethasone has a modulatory effect on nerve block pharmacodynamics rather than its action being restricted to a single local anesthetic. Proposed mechanisms include dexamethasone induced vasoconstriction, which may slow the absorption of local anesthetics (21); suppression of the synthesis and secretion of inflammatory mediators (22); and reduced transmission in unmyelinated C fibers (23).

A distinctive component of our research was the investigation of associations between hemodynamic parameters and analgesic duration. Weak correlations were observed between intraoperative and postoperative heart rates, oxygen saturation, systolic blood pressure, and duration of analgesia. These associations involved both intra and postoperative vital signs and the timing of first analgesic medication intake. These results should be interpreted with caution, as the exploratory design and limited sample size preclude causal inference. Nevertheless, the findings highlight the need for further research and provide preliminary insights into factors that may influence anesthetic effectiveness in specific populations.

Several limitations of this study should be acknowledged. Pain assessment was restricted to the early postoperative period to evaluate block related analgesia specifically, the primary outcome. Longer term assessment could provide additional information on delayed pain patterns and neurological outcomes, representing a limitation of this study. Future investigations with extended follow up and inclusion of functional outcomes, such as facial swelling and maximum mouth opening, are warranted.

## Conclusions

The addition of dexamethasone to articaine significantly prolonged analgesia after inferior alveolar nerve block in patients undergoing mandibular third molar surgery. Larger studies with extended follow up are required to elucidate the precise mechanism of action, establish the optimal dosing strategy, and confirm the safety profile before dexamethasone can be routinely recommended as an adjuvant in local anesthetic solutions.

## Figures and Tables

**Table 1:** Table Comparison of baseline characteristics and analgesia-related parameters among the three groups.

	Groups			
	Group A	Group B	Group C	p
Age (Mean±SD)	24.6±5.6	26.1±5.3	24.8±4.9	0.647
Gender (%)				
Male / Female	9 (45.0)/11 (55.0)	10 (50.0)/10 (50.0)	9 (45.0)/11 (55.0)	0.935
Duration of analgesia (Mean±SD)	174.3±15.8	210.9±23.7	260.9±25.3	<0.001
Duration of operation (Mean±SD)	24.1±5.6	21.2±7.3	21.4±7.8	0.304
Amount of the analgesic consumed in the first 24 hours (median [IQR])	2.0 [1.8-2.0]	2.0 [2.0-2.0]	2.0 [1.8-2.0]	0.856
Amount of the analgesic consumed in 24 hours after the onset of pain (median [IQR])	2.0 [1.8-2.0]	2.0 [2.0-2.0]	2.0 [1.8-2.0]	0.660
Time of the onset of pain (median [IQR])	7.0 [6.0-9.5]	10.0 [8.0-13.0]	9.0 [7.0-13.0]	0.258
First time of taking an analgesic (median [IQR])	8.5 [7.0-13.0]	9.0 [7.0-10.0]	10.0 [8.0-13.8]	0.332

IQR; interquartile range, SD; standart deviation.

**Table 2:** Table The associations of pre-, intra- and postoperative vital signs with VAS scores among the three groups.

			Groups			
	All Patients	p	Group A (n=20)	Group B (n=20)	Group C (n=20)	Interaction p
SBP		<0.001				0.132Ω
PreOP	116.8 (7.0)		116.2±7.5	117.5±6.0	116.8±7.6	
IntraOP	116.5 (6.6)		116.5±7.2	116.8±6.0	116.2±7.0	
PostOP	116.0 (6.1)		116.2±6.2	116.2±6.0	115.5±6.4	
DBP		0.410				0.159Ω
PreOP	75.8 (5.8)		76.6±6.3	75.3±5.6	75.3±5.8	
IntraOP	75.9 (5.5)		76.5±6.1	75.8±5.1	75.5±5.5	
PostOP	75.7 (5.2)		76.0±5.3	75.7±5.2	75.5±5.5	
Heart Rate		<0.001				0.416Ω
PreOP	76.5 (6.2)		74.5±6.0	77.8±4.9	77.3±7.3	
IntraOP	87.3 (7.4)		84.5±7.1	88.0±5.8	89.2±8.6	
PostOP	79.5 (5.9)		77.6±6.1	80.2±4.4	80.6±6.9	
Saturation		<0.001				0.379Ω
PreOP	99.2 (0.4)		99.1±0.3	99.2±0.4	99.4±0.5	
IntraOP	99.2 (0.4)		99.1±0.2	99.3±0.4	99.2±0.4	
PostOP	99.5 (0.5)		99.4±0.5	99.5±0.5	99.6±0.5	
VAS		0,014				0.609†
PreOP	5.0 [2.0, 7.2]		4.5 [2.0, 9.2]	5.0 [2.8, 7.2]	5.0 [2.0, 7.0]	
PostOP	5.0 [3.0, 8.0]		5.0 [3.0, 9.0]	5.5 [3.0, 7.2]	5.0 [2.0, 7.0]	

Intraop: Intraoperative, preop: Preoperative, postop: Postoperative, VAS: Visual analogue score. Ω: Repeated measures two way. ANOVA: Test was used. Descriptive statistics were given as mean±standart deviation. The p-values those were bold considered as statistically significant (p<0,05).†An R software package npaRLD (nonparametric analysis of longitudinal data in factorial experiments). Descriptive statistics were given as median [IQR].

**Table 3:** Table Correlation of duration of analgesia with preoperative, intraoperative and postop patient parameters.

				Group					
		All Patients		A		B		C	
		r	p	r	p	r	p	r	p
Duration of analgesia	PreOP SBP	0.094	0.474	-0.250	0.287	-0.006	0.979	0.492	0.028
Duration of analgesia	IntraOP SBP	0.049	0.713	-0.266	0.256	0.009	0.971	0.463	0.040
Duration of analgesia	PostOP SBP	0.021	0.873	-0.273	0.244	0.005	0.983	0.454	0.044
Duration of analgesia	PreOP DBP	-0.051	0.698	-0.387	0.092	-0.046	0.846	0.411	0.072
Duration of analgesia	IntraOP DBP	-0.062	0.637	-0.412	0.071	-0.047	0.846	0.326	0.161
Duration of analgesia	PostOP DBP	-0.036	0.784	-0.468	0.037	-0.067	0.780	0.326	0.161
Duration of analgesia	PreOP Heart Rate	0.229	0.078	0.120	0.613	0.217	0.358	0.150	0.528
Duration of analgesia	IntraOP Heart Rate	0.288	0.026	-0.145	0.542	0.029	0.903	0.377	0.102
Duration of analgesia	PostOP Heart Rate	0.260	0.045	0.045	0.852	0.280	0.232	0.203	0.390
Duration of analgesia	PreOP Saturation	0.203	0.120	-0.100	0.674	0.143	0.547	-0.07	0.768
Duration of analgesia	IntraOP Saturation	0.231	0.076	0.138	0.563	0.389	0.09	-0.094	0.695
Duration of analgesia	PostOP Saturation	0.292	0.024	0.296	0.205	0.199	0.401	0.157	0.508
Duration of analgesia	PreOP VAS	-0.138	0.294*	-0.211	0.371*	-0.289	0.216*	0.065	0.786*
Duration of analgesia	PostOP VAS	-0.170	0.193*	-0.234	0.320*	-0.270	0.250*	0.039	0.871*
Duration of analgesia	Age	-0.049	0.709	0.274	0.243	-0.386	0.093	-0.071	0.768
Duration of analgesia	Duration of Operation	-0.135	0.304	0.590	0.006	-0.089	0.708	-0.24	0.309
Duration of analgesia	Amount of the analgesic consumed in the first 24 hours	0.026	0.841*	-0.086	0.72*	-0.050	0.835*	0.161	0.498*
Duration of analgesia	Amount of the analgesic consumed in 24 hours after the onset of pain	0.042	0.748*	-0.086	0.720*	0.052	0.828*	0.161	0.498*
Duration of analgesia	Time of the onset of pain	0.073	0.634*	-0.116	0.68*	-0.079	0.764*	-0.341	0.254*
Duration of analgesia	First time of taking an analgesic	0.03	0.823*	-0.124	0.601*	-0.048	0.841*	-0.33	0.156*

DBP: Diastolic blood pressure, intraop: Intraoperative, preop: Preoperative, postop: Postoperative, SBP: Systolic blood pressure, VAS: Visual analogue score. *: Spearman Rho Correlation coefficient.

## Data Availability

Declared none.
